# Cerebellar Contribution to Motor and Non-motor Functions in Parkinson's Disease: A Meta-Analysis of fMRI Findings

**DOI:** 10.3389/fneur.2020.00127

**Published:** 2020-02-27

**Authors:** Linda Solstrand Dahlberg, Ovidiu Lungu, Julien Doyon

**Affiliations:** ^1^Department of Neurology & Neurosurgery, McConnell Brain Imaging Centre, Montreal Neurological Institute, McGill University, Montreal, QC, Canada; ^2^Department of Psychiatry, University of Montreal, Montreal, QC, Canada; ^3^Functional Neuroimaging Unit, Centre de Recherche de l'Institut Universitaire de Gériatrie de Montréal, Montreal, QC, Canada

**Keywords:** Parkinson's disease, fMRI, motor, cognition, symptoms, meta-analysis

## Abstract

**Background:** Parkinson's disease (PD) results in both motor and non-motor symptoms. Traditionally, the underlying mechanism of PD has been linked to neurodegeneration of the basal ganglia. Yet it does not adequately account for the non-motor symptoms of the disease, suggesting that other brain regions may be involved. One such region is the cerebellum, which is known to be involved, together with the basal ganglia, in both motor and non-motor functions. Many studies have found the cerebellum to be hyperactive in PD patients, a finding that is seldom discussed in detail, and warrants further examination. The current study thus aims to examine quantitively the current literature on the cerebellar involvement in both motor and non-motor functioning in PD.

**Methods:** A meta-analysis of functional neuroimaging literature was conducted with Seed-based D mapping. Only the studies testing functional activation in response to motor and non-motor paradigms in PD and healthy controls (HC) were included in the meta-analysis. Separate analyses were conducted by including only studies with non-motor paradigms, as well as meta-regressions with UPDRS III scores and disease duration.

**Results:** A total of 57 studies with both motor and non-motor paradigms fulfilled our inclusion criteria and were included in the meta-analysis, which revealed hyperactivity in Crus I–II and vermal III in PD patients compared to HC. An analysis including only studies with cognitive paradigms revealed a cluster of increased activity in PD patients encompassing lobule VIIB and VIII. Another meta-analysis including the only 20 studies that employed motor paradigms did not reveal any significant group differences. However, a descriptive analysis of these studies revealed that 60% of them reported cerebellar hyperactivations in PD and included motor paradigm with significant cognitive task demands, as opposed to 40% presenting the opposite pattern and using mainly force grip tasks. The meta-regression with UPDRS III scores found a negative association between motor scores and activation in lobule VI and vermal VII–VIII. No correlation was found with disease duration.

**Discussion:** The present findings suggest that one of the main cerebellar implications in PD is linked to cognitive functioning. The negative association between UPDRS scores and activation in regions implicated in motor functioning indicate that there is less involvement of these areas as the disease severity increases. In contrast, the lack of correlation with disease duration seems to indicate that the cerebellar activity may be a compensatory mechanism to the dysfunctional basal ganglia, where certain sub-regions of the cerebellum are employed to cope with motor demands. Yet future longitudinal studies are needed to fully address this possibility.

## Introduction

Parkinson's disease (PD) is a neurodegenerative movement disorder characterized by classic symptoms including tremor, bradykinesia, rigidity, akinesia, postural instability, and balance problems. Its diagnosis is mainly made through the careful assessment of these symptoms, which become the target of subsequent treatment interventions. However, non-motor functions comprising cognitive, sensory, sleep, emotional, and social abilities are also affected by the disease [for a review, see (1–3)] and may even precede the appearance of the motor symptoms (4). Furthermore, even though the non-motor symptoms can be more detrimental to patients' quality of life than the motor signs (5), they have not yet received the same amount of attention in clinical and research settings alike.

A plethora of studies have established that the neurological underpinnings of PD are tied to the neurodegeneration of the basal ganglia, more specifically the dopaminergic cells of the substantia nigra *pars compacta*. The traditional model of PD states that such a dopaminergic denervation leads to hyperactivity in basal ganglia output nuclei (globus pallidus *internus* and substantia nigra, *pars reticulata*), hence resulting in increased inhibition from thalamocortical and brain stem motor regions, which subsequently leads to impaired movements (6, 7). Indeed, several models have been proposed that discuss how basal ganglia dysfunction has cascading effects on interconnected circuits, including the thalamus and cortical (motor) regions that result in some of the characteristic motor symptoms seen in PD [for an overview, see (8)]. Yet, whether these effects indicate the spreading of the underlying pathology into the non-affected areas, or an adaptive/compensatory response to the basal ganglia neurodegeneration is largely unknown.

Furthermore, although basal ganglia dysfunction can explain many of the motor symptoms seen in PD, it does not adequately explain the non-motor symptoms of the disease, hence suggesting that other brain structures, and the cerebellum in particular, may also be involved in the pathophysiological process. In fact, several lines of evidence support this notion. First, certain PD motor symptoms, like tremor, have been linked to abnormal functional connectivity between the basal ganglia and the cerebellum, via the thalamus (9, 10). Second, despite the fact that the cerebellum has traditionally been considered to play a merely supporting role in motor functioning, adjusting and fine-tuning movements based upon an internal model (11) as well as through a feedforward system (12), it has recently been suggested that the cerebellum is involved in monitoring performance for several types of behaviors (13). To this effect, early cerebellar lesion and neuroimaging studies have linked the cerebellum to a wide range of higher cognitive functions, such as working memory, executive functioning, planning, set shifting and more (14, 15). These findings have been further confirmed and expanded through reports that the cerebellum also plays a role in pain, mood disorders and emotional processing, sensorimotor integration, as well as language and learning (16–20). Finally, investigations in healthy individuals have revealed that the basal ganglia and cerebellum are working synergistically to produce efficient motor and non-motor functioning (19). For instance, both sub-cortical structures are implicated in reinforcement learning and reward (18, 21), motor planning and action understanding (22, 23), as well as sensorimotor prediction and control (24, 25) amongst others. Thus, together these findings likely suggest that the cerebellum is instrumental in non-motor symptoms in PD. Indeed, a recent meta-analysis on volumetric cerebellar changes in neurodegenerative disorders proposed that the cerebellum plays a bigger role in cognitive, than in motor symptoms experienced by PD patients (26). Neuroimaging studies have also been consistent with this notion, as positron emission tomography studies using ^18^F-fluorodesoxyglucose have reported increased metabolism in the cerebellum to be linked to cognitive impairment in PD patients, hence characterizing the observed cerebellar hypermetabolism as a part of a “PD related cognitive pattern” (27–30) that is not modulated by treatment interventions (27). There are also increasing amounts of evidence from functional magnetic resonance imaging (fMRI) studies, which support the notion of aberrant activity in the cerebellum of PD patients during both task and rest conditions (31–35).

Despite the recent advances described above, however, there are several important factors that limit our understanding of the role of cerebellum in PD. First, in most imaging studies with PD patients, the cerebellum is commonly reported to be active in response to non-motor paradigms; yet its activation is rarely discussed in detail or seldom constitutes the focus of the study. Moreover, even though anatomical boundaries of cerebellar regions have been clearly defined with specialized functional topography (36, 37) and atlases are readily available (38, 39), the findings are commonly described in the context of the cerebellum as a whole, without reference to its sub-regions. It is therefore not clear whether certain parts of the cerebellum are more implicated than others in relation to motor and non-motor functioning in PD. Finally, the potential role of the cerebellum in PD has been discussed elsewhere in narrative reviews (40, 41), but the cerebellar involvement remains largely unclear, especially in regards to the pathological and/or compensatory mechanisms at play. With exception of one meta-analysis of cerebellar gray matter atrophy across several neurodegenerative conditions (that did not report any findings in PD patients) (26), the existing literature lacks a quantitative and systematic review of cerebellar findings in PD based upon functional neuroimaging methods. In response to this knowledge gap, the current systematic review and meta-analysis appraises the fMRI literature on cerebellar involvement in both motor and non-motor processes in patients with PD. First, a general analysis including all fMRI studies comparing task-related activity in PD vs. matched control participants is carried out, before stratification of motor and non-motor studies, which are then examined separately in order to determine whether certain regions of the cerebellum are specifically implicated in these functions. Relationships with disease severity and duration are also assessed. With this, we aim to develop a greater insight into the role of the cerebellum in PD, with a particular focus on its involvement in motor and non-motor functioning.

## Methods

### Study Eligibility and Research Methods

An extensive search was carried out on Pubmed, and included the following search terms:

- “Parkinson's Disease” [AND] “functional magnetic resonance imaging” [AND] cerebellum
- “Parkinson's Disease” [AND] “fmri” [AND] cerebellum
- “Parkinson's Disease” [AND] “fmri”
- “Parkinson's Disease” [AND] “functional magnetic resonance imaging”.

**Figure 1:**
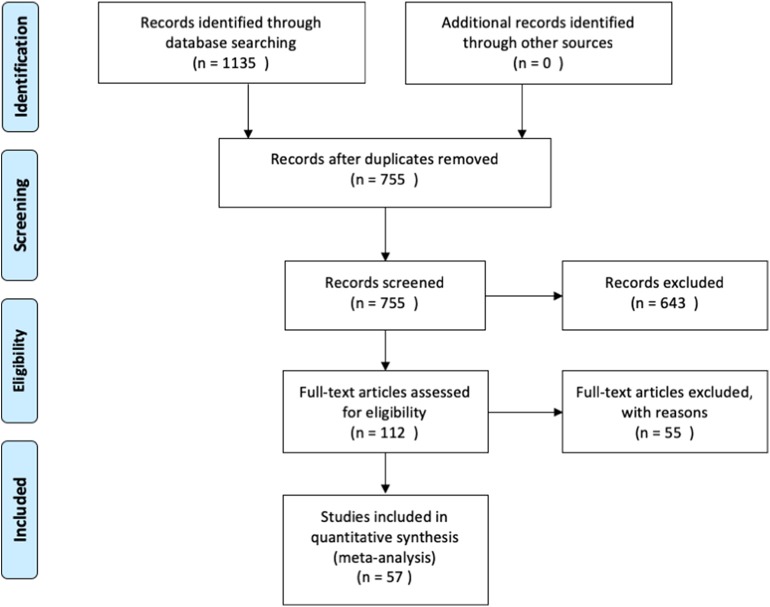
Overview of the study identification and selection process.

We then used the following inclusion criteria for the selection of eligible studies. They had to: (1) be published in peer-reviewed journals, written in English and not behind paywalls that were not covered by McGill University Library subscriptions; (2) include a healthy control group that was compared with PD patients; (3) assess functional brain activity with fMRI in response to a task paradigm; and (4) include results from original research, not from secondary sources (i.e., reviews). The meta-analysis was conducted in accordance with the Preferred Reporting Items for Systematic Reviews and Meta-Analysis (PRISMA) statement (see Figure 1 for overview, and Supplementary Materials for the PRISMA checklist). The last search was conducted on December 9th, 2019.

### Meta-Analysis

Cerebellar activation coordinates and the effect size from comparisons between PD and a control group in response to motor or cognitive paradigms were first extracted from each paper, together with scanning and preprocessing parameters. The meta-analysis was carried out using seed-based mapping (SDM) [(42), https://www.sdmproject.com], a software that conducts meta-analyses similarly to the activation likelihood estimation, and multilevel kernel density analysis approaches, but with integrated sensitivity analyses, effect size estimation, as well as the option to include negative and nil-findings. Data were then preprocessed to achieve a voxelwise recreation of the studies using an isotropic Full Width Half Maximum (FWHM) of 20 mm, and a voxel size of 2 mm^3^. Third, the global means of all studies were analyzed (the meta-analysis) using 50 imputations, creating beta-coefficients and a mean activation map including the associated variance. Finally, the maps were corrected for multiple comparisons with Family Wise Error (FWE) using 500 permutations. However, as corrected maps were not sensitive enough to detect clusters associated with group differences, an uncorrected *p* > 0.005 with an extent threshold of >10 voxels was later used for the meta-analyses.

Data preprocessing included information on coordinates space (MNI or Talairach) and on the analysis package used (SPM, FSL or “other”). Each study's t-threshold was included in the analyses as a measure of the statistical threshold used for the findings reported in each study. In cases where this was not stated in the paper, SDM's built-in effect-size estimation tool was used to provide effect-size threshold estimates. Information on subjects' age, gender ratio, as well as information on the patients' UPDRS III, Hoehn & Yahr scores and disease duration was extracted from the papers whenever available (see Table 1). The disease duration and UPDRS scores were later used for correlation analyses in the meta-analysis.

**Table 1:** Study demographics of all included studies.

**References**	**PD (n)**	**HC (n)**	**f/m PD**	**f/m HC**	**PD age (±SD)**	**HC age (±SD)**	**UPDRS III**	**Hoehn & Yahr**	**Disease duration (years)**	**Nil-finding**	**Task**
Vriend et al. (43)	21	37	4/10	8/6	59.0 (±10.4)	56.2 (±9.9)	21.6 (±8.3)	1.9 (±0.4)	0.19 (±0.31)	No	Cognitive control task
Filip et al. (44)	21	28	10/11	14/14	68 (±4.85)	66.4 (±6.9)	Not stated	2.24 (±0.57)	7.69 (±3.8)	No	Cognitive: impulse control
Heller et al. (45)	26	25	1/11	5/7	63.9 (±8.4)	62.6 (±9.0)	26.8 (±11.6)	2	8.18 (±6.83)	No	Emotional processing
Yu et al. (31)	8	8	6/14	10/10	59.4 (±8.4)	59.5 (±9.5)	31.1 (±10.8)	Not stated	5.9 (±2.6)	No	Motor and auditory task
Martin et al. (46)	22	22	4/10	5/9	52.5 (±10.7)	48.5 (±12.4)	15.6 (±6.4)	1.4 (±0.6)	Not stated	No	Motor and planning task
Burciu et al. (47)	20	20	1/8	5/10	65.8 (±8.0)	64.8 (±8.8)	31.9 (±9.6)	2.0 (±0.3)	4.14 (±1.65)	No	Motor and visual
Rottschy et al. (48)	23	23	8/14	8/14	67.2 (±6.2)	65 (±4.4)	23.9 (±16.1)	1.5 (±0.9) (ON)	4.7 (±4.2)	No	Motor and working memory task
Pinto et al. (49)	9	15	7/13	5/5	59 (±9)	55 (±11)	33 (±13)	Not stated	14 (±7)	No	Motor hand movement and speech
Caproni et al. (50)	11	11	3/8	3/8	65 (±4.98)	65.1 (±5.86)	20 (±4.5)	2	3.8 (±1.5)	No	Motor task
Husárová et al. (51)	20	21	9/11	10/11	55.4 (±9)	57 (±7.3)	18.08 (±3.8)	Not stated	2.5	No	Motor task
Poisson et al. (52)	6	10	2/4	6/4	65 (±10)	53.6 (±8.5)	16 (±5.1)	Not stated	5.4 (±4.6)	No	Motor task
Wu and Hallet (33)	12	14	4/8	4/8	61.2 (±7.64)	61.8 (±no SD)	25.5 (±7.4)	2.04 (±0.62)	6.33 (±2.84)	No	Motor task
Jia et al. (53)	22	22	8/14	8/14	61.04 (±4.38)	60.59 (±4.64)	16.45 (±4.63)	1.64 (±0.44)	4.04 (±1.81)	No	Motor task
Toxopeus et al. (54)	12	18	5/7	9/9	59 (±9)	58.7 (±5.4)	22 (±7)	2.0 (±0.5)	6 (±4)	No	Motor task
Planetta et al. (55)	14	14	7/12	17/5	64 (±8.7)	61.9 (±8.4)	29.6 (±5.3)	Not stated	5.9 (±5.5)	No	Motor task
Neely et al. (56)	14	14	5/5	6/5	64.0 (±8.7)	60.2 (±9.2)	29.6 (±5.3)	Not stated	Not stated	No	Motor task
Cerasa et al. (57)	10	11	8/12	10/10	64.2 (±13.6)	63.4 (±9.3)	27.5 (±8.8)	2.5 (±0.6)	7.2 (±3.5)	No	Motor task
Schwingenschuh et al. (58)	20	10	5/7	9/9	66.8 (±7.2)	33.9 (±8.9)	37.9 (±11.1)	2.2 (±0.4)	6.3 (±3.1)	No	Motor task
Kraft et al. (59)	12	12	5/10	5/10	60.8 (±7.3)	53.0 (±12.0)	21.0 (±3.3)	1.8 (±0.5)	3.1 (±1.1)	No	Motor task
van der Stouwe et al. (60)	12	18	7/9	8/7	59 (±9)	59 (±5)	21.5 (±6.9)	1.9 (±0.5)	Not stated	No	Motor task
Wu et al. (61)	15	15	Not stated	Not stated	59.73 (±8.27)	60.3	20.67 (±3.48)	1.7 (±0.37)	3.47 (±1.6)	No	Motor task
Wurster et al. (62)	10	10	7/14	7/12	66.4 (±7.2)	64.9 (±8.14)	20.7 (±9.1)	2 (±0.83)	6 (±5.6)	No	Motor task
Hughes et al. (63)	16	15	11/10	11/11	63.9 (±7.5)	66.5 (±5.9)	31.3 (±11.)	2.0 (±0.5) (ON)	7.6 (±3.7)	No	Motor task
Lemos et al. (64)	19	22	6/15	16/21	64.9 (±6.3)	66.4 (±9.5)	Median: 19 (±19)	Median: 1.5 (±1)	4 (±8)	No	Saccade task
Takeda et al. (65)	9	7	5/4	5/2	54	51	Not stated	2.2	Not stated	No	Sensory: olfactory
Tessitore et al. (66)	20	18	9/11	8/10	60 (±8.9)	55.9 (±5.2)	10.1 (±7)	1.4 (±0.5)	1.2 (±0.5)	No	Sensory: pain
Harrington et al. (67)	21	19	7/5	5/7	67 (±9.4)	64.6 (±8.5)	29.6 (±10.4)	2	Not stated	No	Working memory task
Snijders et al. (68)	24	21	3/9	6/10	60.2 (±8.9)	57.0 (±9.1)	31.6	Not stated	8.45	Yes	(Imagined) motor task
Maidan et al. (69)	20	20	8/16	4/6	72.9 (±1.6)	69.7 (±1.3)	29.8 (±2.4)	Not stated	6.8 (±1.3)	Yes	(Imagined) motor task
Baglio et al. (70)	15	11	4/11	7/4	66.5 (±6.4)	66.9 (±5.7)	21.5 (±7.24)	1.56 (±0.46)	Not stated	Yes	Cognitive: attention and inhibition
Labudda et al. (71)	10	12	2/10	6/6	57.6 (±7.83)	62.33 (±4.81)	Not stated	3	7.1 (±3.7)	Yes	Cognitive: decision
Gescheidt et al. (72)	18	18	4/14	7/11	52.67	50.61	18.89	1.97	6.33	Yes	Cognitive: decision
Schonberg et al. (73)	7	17	5/2	13/4	58.7 (±3.7)	60 (±4.1)	12.4 (±7.2)	1.9 (±0.7)	4 (±2.9)	Yes	Cognitive: error detection
Grossman et al. (74)	7	9	Not stated	Not stated	71 (±10.2)	65.7 (±10.2)	Not stated	1	Not stated	Yes	Cognitive: sentence comprehension
Ibarretxe-Bilbao et al. (75)	24	24	8/16	8/16	56.13 (±8.5)	57.58 (±8.9)	14.67 (±3.5)	1.73 (±0.4)	3.06 (±1.6)	Yes	Cognitive: speech
Isaacs et al. (76)	13	18	7/6	12/6	62.23 (±6.83)	68.06 (±9.52)	Not stated	1.46 (±0.52)	5.39 (±3.8)	Yes	Cognitive: speech
Sachin et al. (77)	8	6	3/8	4/8	Not stated	Not stated	Not stated	Not stated	Not stated	Yes	Cognitive: speech
Nemcova et al. (78)	16	55	4/75	38/17	62.7 (±6.8)	66.7 (±7.3)	16.8 (±9.1)	Not stated	4.4 (±2.5)	Yes	Cognitive: visual object-matching task
Dan et al. (79)	25	32	10/15	17/15	64.7 (±8.3)	63.3 (±7.7)	30.4 (±11.1)	2 (±0.5)	11.9 (±4.7)	Yes	Emotional recognition task
Pohl et al. (80)	13	13	5/8	6/7	Median: 68	Median: 65	24.21 (±9.60)	Not stated	5.94 (±4.39)	Yes	Emotional recognition task
Nombela et al. (81)	10	10	9/14	10/13	60.5 (±3.45)	59.6 (±4.47)	22.2 (±7.9)	2.5 (±0.5)	8.1 (±2.0)	Yes	Executive functioning task
Rowe et al. (82)	12	12	9/15	9/12	62 (±6)	62 (±6)	33.7 (±8.54)	2.46 (±0.45)	5.4 (±3.6)	Yes	Motor and attention task
Arnold et al. (83)	20	20	8/12	8/12	63.9	64.2	26.1	1.65	5.8	Yes	Motor and cognition task
Nieuwhof et al. (84)	19	26	4/15	10/16	70.7 (±6.1)	71.2 (±5.3)	36.0 (±8.2)	2	6.2 (±4.8)	Yes	Motor and cognition task
Zhao et al. (85)	21	22	Not stated	Not stated	60.43 (±9.65)	59.23 (±11.12)	20.57 (±3.83)	1.2 (±0.3)	1.95 (±1.8)	Yes	Motor and sensory task
Sabatini et al. (86)	6	6	2/4	2/4	61 (±8)	59 (±19)	16 (±4)	2.7 (±0.5)	5 (±2)	Yes	Motor task
Matt et al. (87)	13	14	6/7	5/9	58.7 (±13)	57.4 (±9.8)	30.2 (±12.2)	2.35 (±0.32)	6.3 (±4.7)	Yes	Motor task
Tessa et al. (88)	11	10	2/9	3/7	68 (±8)	64 (±3.8)	13.5 (±4.8)	1.2 (±0.3)	1.5 (±0.5)	Yes	Motor task
Hughes et al. (89)	20	20	10/10	13/7	65.5	65.2	22.2	2.2	Not stated	Yes	Motor task
Yan et al. (90)	11	12	0/26	0/25	61.5 (±7.1)	65.5 (±10.1)	20.1 (±6.3)	Not stated	4.9 (±3.9)	Yes	Motor task
van Eimeren et al. (91)	20	10	9/11	'5/5	50.3 (±7.8)	50 (±8.7)	21.95 (±13.6)	Not stated	10.86 (±7.69)	Yes	Motor task
Spraker et al. (92)	14	14	'6/8	(mached)	57.2 (±9.6)	57.6	18 (±8.1)	1.7 (±0.45)	16.5 (±10.8)	Yes	Motor task
Bedard et al. (34)	10	10	'5/5	8/2	57.4 (±8)	62.4 (±10)	14 (±7.8)	Not stated	Not stated	Yes	Motor, sensory- and learning
Westermann et al. (93)	12	16	5/5	'6/4	57.1 (±2.2)	64.7 (±1.4)	Median: 28	Median: 2	Median: 3.3	Yes	Sensory (olfactory) task
Lefebvre et al. (94)	34	17	12/23	7/10	63.1	62.76	23.4	2 (±0.83)	8.53	Yes	Sensory (visual) task
Caminiti et al. (95)	13	12			63.3 (±6.3)	59 (±2.3)	NA	1-2	5 (±3.4)	Yes	Working memory task
Simioni et al. (96)	19	20	0/10	0/10	66 (±8.6)	65 (±6.7)	15.3 (±5.4)	2.4 (±0.7)	6.9 (±3.3)	Yes	Working memory task

*F, female; HC, healthy controls; M, male; PD, Parkinson's Disease; UPDRS, Unified Parkinson's Disease Rating Scale*.

One of the benefits of our method (SDM, described above) is the option to include studies with nil-findings. These studies were included in the main analysis as studies with no peaks, allowing us to increase accuracy of our analysis. In studies where neither Z-scores, nor F-values were reported in the between groups comparisons, the SDM's conversion tool was utilized to obtain the corresponding t-statistic. As the effect size was not given in a few of the articles, the peaks were then marked as “positive” or “negative,” hence denoting direction of the contrast used. Because of the variability in study methodologies, and to include as much data points as possible, studies utilizing an ROI approach were also included in the analysis, even though in literature, the cerebellum may be an uncommon ROI.

The findings were then inspected for heterogeneity and bias by examining the peak values and the corresponding I^2^ statistic and its funnel plot. I^2^ is a test of heterogeneity for meta-analytical studies, where a low value generally represents a low level of heterogeneity. Egger statistics were also examined by plotting the effect size against precision of the studies as a measure of publication bias. Finally, the SDM toolbox provided results in MNI coordinate space which were confirmed both with the Diedrichsen probabilistic cerebellar atlas (39) in FSL Eyes (https://fsl.fmrib.ox.ac.uk/fsl/fslwiki/FSLeyes), as well as with Schmahmann et al.'s MRI cerebellum atlas (38).

#### Medication Status

To be as inclusive as possible, studies that included patients who were not asked to refrain from taking medication (i.e., in an ON-state) were also included. Since previous studies have found medication to have a “normalizing” effect on the neural activity of PD patients (97, 98), a separate sensitivity analysis using only patients in their OFF-state (i.e., patients who were asked to stop taking medication for ~12 h), was also carried out.

### Motor Studies

In order to investigate group differences in cerebellar activation(s) specific to motor paradigms, a meta-analysis including only studies using a motor-task was also conducted. As we aimed to localize regions specifically linked to motor functioning in PD patients, only studies with reported differences (i.e., no nil-findings) were thus included in this analysis. The uncorrected threshold was kept at *p* < 0.005 with a cluster size threshold of 10 voxels.

### Cognitive Studies

Likewise, a separate meta-analysis was conducted including only studies employing cognitive paradigms. Here, as before, an uncorrected threshold of *p* < 0.005 was used, with an extent threshold of 10 contiguous voxels.

### Meta-Regression Analyses

Two separate regression analyses were also performed to examine the relationship between the pattern of cerebellar activations and the patient's UPDRS III motor scores, as well as the disease duration. As five studies had not defined whether the UPDRS scores referred to the motor subscale (UPDRS-III) or to the total, we thus conducted a sensitivity analysis including only those that explicitly stated that they used the motor subscale. For exploratory purposes, the threshold used for the meta-regression analyses were kept at a liberal uncorrected *p* < 0.05 and extent threshold of >10 voxels.

Finally, we used PD patients' cognitive status [assessed through scores on cognitive tests, such as the Mini Mental State Examination (MMSE) (99)] in a regression to explore the relationship between this variable and their related cerebellar activity in a meta-regression analysis.

## Results

### Selected Studies

An overview of the study identification, screening and selection process is presented in Figure 1. Our search yielded a total of 755 articles after duplicate findings were removed. After further screening, 112 articles were further assessed yielding 57 articles that fulfilled the inclusion criteria. The total number of subjects across all studies included in this meta-analysis was 1856 (890 PD patients and 966 HC), with an average age of 62.07 (±4.69) and 60.69 (±6.15), respectively. The PD patient sample had an average disease duration of 5.90 (±3.05) years, an average Hoehn & Yahr score of 1.92 (±0.41), and an average UPDRS III score of 23.24 (±6.63). A summary of the study demographics can be found in Table 1. Of the 57 studies, 30 did not report any significant differences between PD and controls in response to either motor or cognitive paradigms. A chi-square test assessing whether the prevalence of cerebellar findings across studies was different from chance (i.e., the null hypothesis being that half the studies will show cerebellar difference and half will not) did not reach significance [χ2_(df = 1)_ = 0.157, *p* = 0.691]. This indicates that—without accounting for any other variable (i.e., type of tasks, medication status, etc.)—the probability of finding differences in cerebellar BOLD activity across fMRI studies comparing PD patients and healthy controls was not different from 50%.

### Meta-Analysis

**Figure 2:**
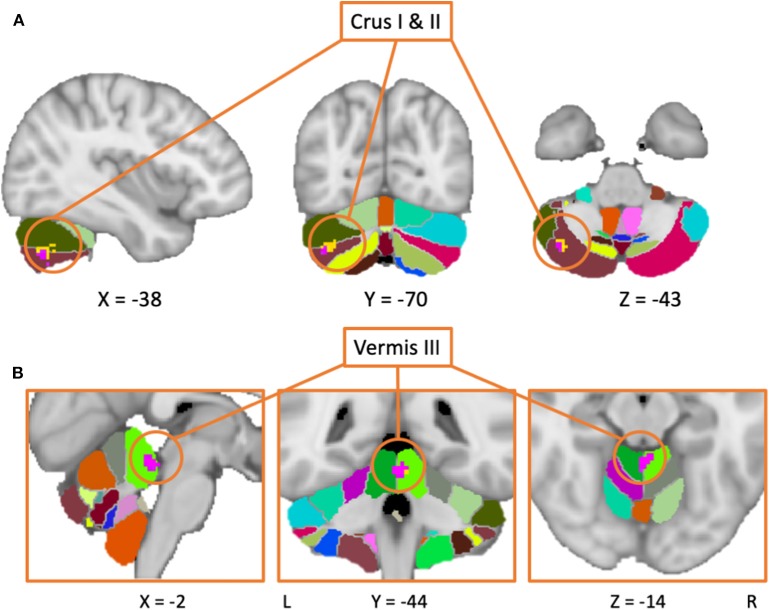
Results from the meta-analysis including both medicated and unmedicated patients (yellow area), as well as the results including only studies with unmedicated patients (purple area) are overlaid on the MNI152 template brain with a probabilistic cerebellar atlas (39). **(A)** The clusters are mainly located in Crus II (in brown), with the larger cluster also expanding into Crus II (in olive) and lobule VI (in light green). **(B)** Both clusters are located in the vermal lobule III. In this case, the cluster from the main meta-analysis (in pink) is overlaid the larger cluster from the second meta-analysis including only patients in the OFF-state (blue cluster).

Despite the fact that 30 of the studies did not report any significant differences between patients and controls in response to tasks, the general meta-analysis of all selected fMRI studies yielded significant results implicating the cerebellum, even after accounting for the nil findings. It revealed two positive activation clusters (i.e., hyperactivation in PD patients as compared to healthy controls), one predominantly covering the left Crus I and Crus II (Table 2, Figure 2) while the other cluster covered largely the vermal area of lobule III. In addition, the results revealed two negative clusters that were located over the fusiform gyrus, and lobule IV/V (Table 2).

**Table 2:** Results from the meta-analyses and meta-regression with their corresponding coordinates and regions.

	**Region**	**Hemisphere**	***x***	***y***	***z***	**SDM-Z**	***p*-value**	**Voxels**	**Peak I^**2**^**	**Eggers bias**	**Eggers *p*-value**
All studies (*n* = 57)	Crus II	L	−38	−70	−42	3.123	0.000894606	66	43.397	0.74	0.530
	Ver III/IV	L	−2	−42	−16	2.891	0.001919806	18	38.690	0.21	0.856
	**Negative clusters:**										
	Fusiform gyrus	R	30	−44	−18	−2.844	0.002227843	13	27.541	−0.66	0.530
	**Local peaks:**										
	Fusiform gyrus	R	30	−44	−18	−2.844	0.002227843				
	Lobule IV/V	R	26	−46	−22	−2.686	0.003616929				
	Lobule IV/V	R	20	−50	−28	−3.038	0.001190126	10	67.158	−3.51	0.001
OFF-state (*n* = 36)	Ver III/IV	L/bilateral	−2	−44	−14	3.332	0.000430882	59	6.574	0.68	0.635
	**Local peaks:**										
	Ver III	L/bilateral	−2	−42	−14	3.332	0.000430882				
	Lobule IV/V	L	−6	−54	−8	2.878	0.002003968				
	Crus II	L	−40	−66	−48	3.127	0.000883460	20	15.949	0.69	0.643
	**Negative clusters:**										
	Lobule IV/V	R	16	−50	−22	−3.040	0.001183808	30	42.218	−1.43	0.323
Cog studies (*n* = 5)	Lobule IV/Lobule III/Ver III	R	10	−44	−18	3.647	0.000132442	158	8.546	2.28	0.843
	**Local peaks:**										
	Lobule IV/Lobule III/Ver III	R	10	−44	−18	3.647	0.000132442				
	Lobule I-IV	R	8	−38	−26	3.575	0.000175357				
	Lobule I-IV	R	6	−38	−14	3.440	0.000290871				
	Lobule VIII	L	−32	−60	−48	3.027	0.001234889	88	9.701	7.87	0.353
	**Local peaks:**										
	Lobule VIII	L	−32	−60	−48	3.027	0.001234889	32			
	Lobule VIII	L	−18	−64	−46	2.995	0.001370609				
	Lobule VIII	L	−24	−64	−48	2.942	0.001628339				
	Lobule VIII	L	−18	−68	−48	2.942	0.001628876				
	Lobule VI/V	L	−20	−74	−18	2.891	0.001922667	11	10.196	9.66	0.229
	**Local peaks:**										
	Lobule VI	L	−20	−74	−18	2.891	0.001922667				
	Crus I	L	−18	−84	−26	2.592	0.004770517				
UPDRS III regression (*n* = 25)	**Negative clusters:**										
	Lobule VI	R	10	−62	−28	−2.312	0.010397077	84	3.767	−0.03	0.979
	Vermal lobule III	Bilateral	2	−74	−34	−2.189	0.014311850	49	28.736	−0.04	0.978

*Local clusters refer to clusters where more than one local peak was identified. Coordinates are in MNI space*.

Of note, almost half of the studies that reported a nil-effect (when comparing patients and controls) included participants that were scanned in the ON medication state (14/30) (status unknown in 2/30), while only three of the studies that reported a between-group effect included patients in the ON state (3/27) (status unknown in 2/27). A chi-square test assessing whether the PD patients medication status (ON vs. OFF) was associated with the prevalence of cerebellar findings across studies was significant [$\chi_{\left(\text{df}=1\right)}^{2}$ = 6.202, *p* < 0.05]. This indicates that one is significantly more likely to find cerebellar differences between PD patients and healthy controls across fMRI studies that included patients in the OFF state, compared to those that involved patients in the ON state. To investigate the effect of medication, a separate analysis was then performed, including only studies where patients were in the OFF-state (36 studies). This analysis yielded two positive and one negative activation clusters similar to those identified in the previous analysis when all studies were included. The largest positive cluster was located in vermal lobule III/IV, although with a smaller spatial extent as compared to the same cluster obtained in the main meta-analysis (i.e., including all studies). The other cluster was located over Crus II with some voxels extending into Crus I. Both of these activation clusters overlapped with those obtained from the main meta-analysis. As with the main analysis, the OFF-state studies also resulted in a negative cluster in lobule IV/V. Figure 2 shows the results of both analyses overlaid on a cerebellar atlas using the MNI template brain.

### Motor Studies

Thirty-one out of 57 studies used motor paradigms to assess differences in patients vs. controls; of these, 20 showed significant differences in cerebellar activation between patients and controls, and were thus included in the analysis. The meta-analysis did not reveal any significant clusters of activation related directly to differences between PD patients and healthy controls during tasks tapping into motor functioning.

### Cognitive Studies

**Figure 3:**
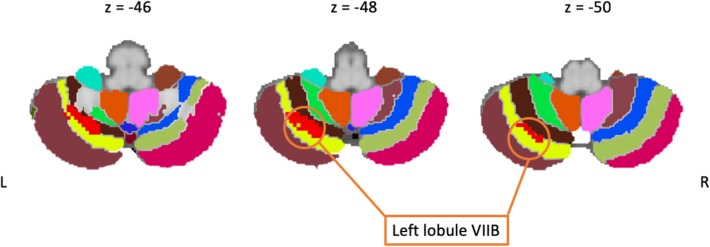
Including only cognitive studies showed an activation cluster (red) over the left lobule VIIB (yellow), here overlaid on coronal slices of the cerebellum.

Twenty-one studies employed a cognitive task. These included paradigms that tested a variety of cognitive functions including: planning (46), cognitive control (43, 44, 70, 72, 73), attention (82), memory and working memory (48, 95, 96), executive functioning (67, 81, 84), object recognition (78), learning (34), imagery (68), decision making (71), linguistic processing (74–76, 83). However, only five studies reported significant differences between patients and controls. The meta-analysis based upon these studies revealed clusters of increased activity in PD patients in the left lobule VIII and VI and in the right lobule IV and V (see Figure 3), indicating that these areas are particularly implicated in cognitive functioning in PD patients.

### Meta-Regressions

#### UPDRS

**Figure 4:**
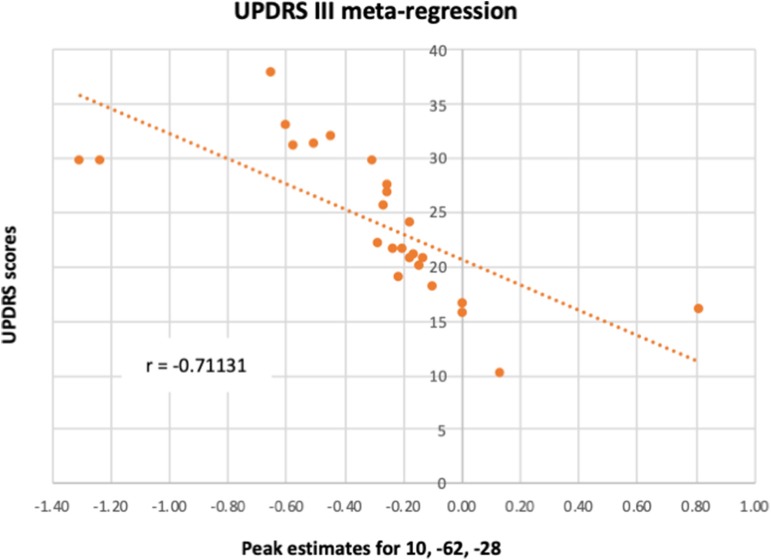
A meta-regression assessed the relationship between PD cerebellar activity in response to all paradigms with patients' UPDRS III scores. The figure illustrates the correlation between UPDRS III scores and each study's estimates of the activity in lobule VI from the local peak in the cluster 10, −62, −28.

A meta-regression was conducted to examine the possible relationship between the pattern of cerebellar activity and the UPDRS scores in PD patients. Overall, the UPDRS scores (100) were listed in 25/27 studies (with nil-finding studies excluded). The meta-regression conducted on these studies revealed a negative correlation between the average UPDRS scores and cerebellar activation in a cluster covering the right lobule VI, suggesting that reduced level of functional activity in this area was particularly linked to the patients' motor symptomology. An additional negative correlation was found in a cluster located within the vermal lobule VIII region. Upon visual inspection, the latter cluster was shown to cover mainly the right vermal VII-VIII, with some bordering voxels in right and left Crus II. Average estimates from each study were extracted from the local peak (*x* = 10, *z* = −62, *y* = −2) and plotted against UPDRS scores, resulting in a significant correlation between the two (*r* = −0.711, *p* < 0.001) (see Figure 4). It is important to note that 20 out of the 25 studies reported explicitly the UPDRS III (i.e., part 3 of the UPDRS test—the motor subscale) scores. For the remaining five studies it was unclear whether the listed UPDRS scores were referring to the total score or to the motor subscale (UPDRS III). As such, we conducted a sensitivity analysis, assessing the impact of the five studies on the UPDRS correlation. When these five studies were excluded from the correlation, the Pearson correlation coefficient remained significant (*r* = −0.728, *p* < 0.001).

#### Disease Duration

Disease duration (in years) was reported in 22/27 studies (with nil-finding studies excluded). The meta-regression examining the relationship between cerebellar functional differences in PD compared to HC during both motor and cognitive paradigms revealed no significant correlation with disease duration.

#### Cognitive Functioning

We examined the reported cognitive scores (either MMSE or MOCA) from each study with the intent of conducting a meta-regression analysis involving the PD patients' level of cognitive functioning and their general pattern of cerebellar activation. The average MMSE scores reported in the 22 studies, in which cognitive measures were stated, was 28.6 (STD:1) (of a total of 30). Most studies used MMSE cut-off scores (most often a cut-off of 26/30) as an inclusion/exclusion criterion in order to avoid including PD patients with cognitive decline, while in some studies the MMSE or Montreal Cognitive Assessment [MoCA (101)] scores were not indicated, hence resulting in a limited spread of the scores in the studies where these were reported. Including cognitive scores in the meta-analysis could therefore have been a source of bias. Consequently, we opted against conducting a meta-regression with these scores.

## Discussion

This is the first meta-analysis of functional neuroimaging studies that aimed to quantify task-related differences in cerebellar neural activity in PD patients relative to healthy controls. Our findings reveal that PD patients showed hyperactivity in Crus I and II, as well as in lobule I—IV, across studies that used both motor and non-motor (i.e., cognitive) paradigms. Furthermore, results from the meta-regressions showed that the functional changes found in cerebellar regions VI, and vermal lobule VII and VIII correlate negatively with UPDRS scores. Together, such findings provide support for the functional specificity of the cerebellum in relation to the disease, as discussed in detailed below.

### Overall Hyperactivation in PD Patients

The meta-analysis including all studies (both motor and non-motor paradigms) revealed a cluster of increased activation over the right Crus I and II in PD patients compared to controls. This pattern was evident even when the analysis was performed using only studies with patients in their OFF-state, hence indicating that this result does not depend on the medication status.

Lobules Crus I and II have been referred to as a part of the “cognitive cerebellum” (37, 102). This notion is based upon the following three lines of evidence; first, from non-human primate studies that found direct anatomical connections to exist between these cerebellar sub-regions and the pre-frontal cortex (103); second, from previous reports in humans using resting state functional connectivity analyses where connectivity between Crus I and II with the dorsolateral pre-frontal cortex (DLPFC) and anterior pre-frontal cortex (APFC) has been observed (26, 37, 104–106); and finally, from a meta-analysis of neuroimaging activation studies on cerebellar functional topography in healthy individuals that provided evidence that Crus I and II are frequently activated during cognitive tasks (107).

Both structural and functional studies in groups of PD patients also support the role of Crus I and II in cognitive functioning. For instance, a meta-analysis on gray matter changes in neurodegenerative disorders reported that PD patients showing evidence of a cognitive impairment most frequently also presented a reduction in cerebellar gray matter, while no atrophy in motor regions of the cerebellum was found (26). Furthermore, another structural study showed that gray matter alterations in Crus I can be used to classify between PD patients and matched control subjects with a 95% accuracy (108). Finally, using fMRI, a study in which healthy controls and PD patients executed a motor timing task requiring cognitive demands, the authors reported increased activation in both Crus I and II in PD compared to controls (31). Together, these findings are thus in accord with our results that Crus I and II are overactive in PD patients compared to controls, and that the cerebellar hyperactivation may be linked to cognitive functioning in PD.

Our meta-analysis included almost twice as many studies that focused on “motor” compared to “cognitive” functions. However, the observed hyperactivation in the “cognitive cerebellum” described above is unlikely to represent pure motor or pure cognitive functioning, but rather a more non-specific overactivity in PD patients compared to controls, irrespective of the task. If we consider that some of the motor studies in the current meta-analysis included also sensory components, i.e., auditory (31), tactile (85), and pain processing (66), the idea that hyperactivity in Crus I and II in PD patients is not strictly linked to motor functioning seems to receive more support. Indeed, the lack of relationship between the UPDRS motor scores and Crus I and II functional activity also supports this notion. Thus, although the underlying basis for this hyperactivity is not easy to pinpoint, one could speculate that it reflects a compensatory response to basal ganglia dysfunction.

Finally, our findings revealed a cluster of hyperactivity on the border of the hemispheric and vermal lobule III area, a region linked to sensorimotor/vestibular function (26, 31). Interestingly, a link between symptoms of ataxia and damage to areas II-V of the cerebellum has previously been reported (109). Thus, given that both disorders are associated with problems of balance and postural instability, this could explain why we observed such overactivity within this sub-region in PD patients. Consistent with this interpretation is also the fact that the same activation clusters were obtained when we included the studies investigating only patients in the OFF state in the analysis.

### Cognitive Paradigms Are Linked to PD Hyperactivity in Lobules VI and VIII

When including only studies that employed cognitive paradigms in the meta-analysis, our results revealed stronger activations in lobule VI and VIII in PD patients as compared to controls. Even though both lobules VI and VIII are thought to be part of the cerebellar homunculus (110) and are considered as “motor lobules” due to their anatomical projections to the motor cortex (111, 112), there is also evidence that they contribute to cognitive functions as well. For instance, these lobules have been found to be involved in executive functioning (107) as well as spatial, language and verbal processing (VI) (107) in healthy individuals through their functional connectivity with the APFC and DLPFC (104, 105). Moreover, reciprocal connections between these regions have been described in non-human primates (103). Taken together, these findings thus provide support to the idea that our results reflect a hyperactivation of lobules VI and VIII in PD in response to the cognitive demands of the task, such as working memory and other executive functions. The meta-analysis also resulted in a cluster of activation over lobules IV/V. Both lobules have functional connections to the sensorimotor cortex (36), while studies in non-human primates have also provided evidence for anatomical projections between these two regions (103). Lobule IV and V moreover show a topographical sensorimotor representation that is frequently activated during sensory or motor engagement (113). A cluster of activity over this area in response to cognitive paradigms is thus not unexpected, as most cognitive paradigms carried out in the scanner also requires sensory and motor processing. Stimuli are normally presented with a visual or auditory modality, prompting a motoric feedback, usually with a hand response. While it is true that, in most of these studies that used cognitive paradigms, the behavioral performance of PD patients was impaired relative to healthy controls, hence suggesting that the cerebellar hyperactivation may reflect functional impairment, there were no reported correlations (either positive or negative) between cerebellar activation and behavioral performance. Therefore, besides concluding that this hyperactivation seems to be in response to the cognitive demands of the tasks, our review of the current neuroimaging evidence cannot provide a proper interpretation of its functional role, nor determine whether the pattern of activity reflects a pathological or compensatory mechanism.

### Motor Paradigms Did Not Reveal Any Significant Group Differences in Cerebellar Activation

Twenty studies were identified using motor paradigms and were included in a separate meta-analysis. However, the latter did not result in any significant clusters of cerebellar activation that would indicate a differential functional involvement of this structure when comparing the PD patients with their healthy counterparts across a variety of motor paradigms. A closer examination of these studies indicates, however, that the lack of a significant group difference may be due to the fact that 12 studies reported cerebellar hyperactivations in PD relative to healthy controls, with 8 studies presenting the opposite pattern. It is also important to note that most of these studies did not report significant differences in motor performance between the two groups (this was, in some cases, by design, because all subjects were trained to reach a certain performance level prior to the fMRI session). Thus, it is possible that differences in motor functioning between PD and controls cannot be linked reliably to specific cerebellar sub-regions at the current time, perhaps due to the variation of tasks utilized by the studies included in the meta-analysis. Yet despite the lack of a significant result in this meta-analysis, several useful observations can be drawn from a descriptive analysis of these 20 studies that employed motor paradigms and nevertheless reported significant group differences at the study level. The first observation is that 9 of the 12 studies that reported cerebellar hyperactivations in PD also used paradigms with rhythmic tapping or sequential movements tasks, with the remaining 3 using motor tasks that had a strong cognitive component to it, such as predictive motor timing (51), controlled thumb pressing movements (31), and center-out step-tracking (60). The second is that of the eight studies that reported hypoactivations, half used grip force tasks and the other half required participants to produce various types of sequential or ballistic movements. As such, we can observe that hyperactivations tend to be associated with tasks that had significant cognitive demands, thus supporting the idea that the hyperactivations seen in our general meta-analysis combining motor and cognitive paradigms are likely to reflect the general cognitive task demands.

### UPDRS III Scores Are Negatively Linked to Activity in Lobule VI and Vermal VII and VIII

The meta-regression revealed a negative relationship in PD patients between the UPDRS scores and the cerebellar activity in lobule VI, Crus II and vermal lobules VII/VIII, hence suggesting that patients with the worse motor clinical states also had reduced activity in these areas. The link between the patients' symptoms measured with the UPDRS III and functional activation in these regions can be explained by the known neuroanatomical connections between the cerebellum and the cerebral cortex. For instance, lobule VI is a part of the cerebellar homunculus and is known to have functional and anatomical connections to the sensorimotor system (26, 37, 114–116). Interestingly, the volume of lobule VI has also been shown to correlate positively with motor functioning (dexterity, grip force, coordination and finger tapping speed) in older adults (117). Similarly, anatomical connections between vermal lobules V—VIIIB and the primary motor cortex have been reported and are hypothesized to play a significant role in both active movements and posture (118). Thus, together, these findings may explain the negative association between the activity in vermal lobules VII and VIII and the UPDRS scores through the connections between these cerebellar regions and the sensorimotor system. If this is the case, then the reduction of functional activity in vermis and lobule VI might be the main cerebellar perpetrators involved with the worsening of motor symptoms.

Although PD motor symptoms tend to worsen as the disease progresses, the meta-regression with disease duration did not reveal any significant association with cerebellar activity using the studies considered in the current meta-analysis. One interpretation of this result could be that the mean disease duration reported in these studies is not a very good proxy of disease severity. Indeed, several factors could explain both intra- and inter-study variability in this regard. For instance, it is expected that patients deteriorate at different rates, are diagnosed at various ages and stages of the disease, and have different lifestyles. Moreover, while the average disease duration in the studies included in the meta-analysis ranged between 0.19 and 6.5 years, most studies included only patients in stages I and II (119). By contrast, if one considers that the average disease duration constitutes an adequate proxy of disease severity, the lack of correlation between it and cerebellar activation could again be interpreted as reflecting a compensatory mechanism of the cerebellum. As such, it is conceivable that this mechanism could be set in effect as early as the basal ganglia ceases to function optimally, and that its effectiveness reaches an asymptote when it is unable to engage more resources. Alternatively and as suggested by Wu and Hallett (41), it is also possible that the cerebellum reaches a compensatory peak activity early in the disease, but wears off as the disease progresses and its efforts become futile. Finally, another reason for the lack of correlation with disease duration is the notion that certain changes in cerebellar activity in PD are more directly related to specific symptoms of the disease (120–123), and even influenced by dopaminergic medication as suggested by Mirdamadi (40). Previous research has shown that tremor dominant PD patients may recruit the cerebellum more so than those who are akinetic/rigid predominant (120), the latter being linked more specifically to vermal dysfunction. Although we do not have information regarding the symptom profile of the patient's included in these studies, a akinetic/rigid predominant representation could explain the negative association between the symptom severity and vermal function. Thus, the finding that the UPDRS III, and not disease duration, was found to be linked with cerebellar hypoactivity could be explained by heterogeneity of patient subtypes recruited in the studies. This could also explain why our meta-analysis yielded, not only a non-specific hyperactivity, but a hypoactivity linked to motor symptom severity as well.

Regrettably, there were insufficient data and little variability regarding the measure of cognitive functioning (MMSE or MoCA scores) to conduct an informative meta-regression to assess the relationship between the level of cerebellar activity and cognitive dysfunction in PD patients. Understandably, many studies used cognition scores as a screening measure in order to exclude patients with signs of significant cognitive dysfunction. Thus, a proper assessment of the relationship between cognitive decline and the cerebellum should be the focus of future research. Interestingly, a previous study examined the neural activity of PD patients with and without mild cognitive impairment (MCI) over time, and found that PD patients with MCI showed increased cerebellar activity (mainly Crus I) in the follow-up test, which was not seen in PD patients without MCI (124). This finding further adds to the involvement of Crus I in cognitive functioning in PD patients, and argues for a progressive involvement of cerebellar activity with cognitive function/dysfunction.

### Limitations

By design, our review focused only on task-related activation studies. As such, we did not cover other functional neuroimaging approaches, such as resting-state, which might have provided additional information. A systematic analysis of the results based on this modality is yet to be done, and could bring valuable insight into the role of cerebellum in PD.

This review, as with most studies on PD, is faced with the problem of heterogeneity across studies, both in terms of experimental paradigms and patient samples. The clinical presentation varies from patient to patient, each presenting with different types of symptoms as well as the level of severity of both motor and non-motor symptoms. Although we made efforts to keep the studies as similar as possible, we could not control for the within-study heterogeneity. Moreover, because we aimed to be as inclusive as possible in our meta-analysis, including studies with and without nil-findings from whole-brain and ROI analyses, we can be more certain that the results obtained from this study reflect true differences. Another challenge when reviewing articles in a research domain like the one discussed here, is the difference in methodology and outcome measures used in the respective studies. This is problematic as it makes study comparisons and interpretations more difficult. Prospective studies should therefore aim to use standardized and/or well-established methods and outcome measurements.

## Conclusions

The current review provides valuable insight into the functional role of the cerebellum in PD, in regards to both motor and non-motor functioning. We were able to quantify the current cerebellar findings from the PD task induced fMRI literature, which revealed that an overall hyperactivity is seen in Crus I and II in response to both motor and non-motor paradigms, whereas hypoactivity in lobule VI and Crus II is linked to motor symptoms. These results suggest that certain cerebellar regions show a special implication in motor and non-motor functioning in PD, and that they are linked to the motoric clinical state, but not disease duration. Moreover, the negative correlation between the UPDRS scores and cerebellar activation in lobule VI, Crus II and vermal lobules VII/VIII, together with the lack of a significant correlation with disease duration, provide support for the view that the pattern of cerebellar activity may represent a compensatory mechanism for the basal ganglia dysfunction, where movement impairments place more demands on certain cerebellar sub-regions than others. However, this hypothesis can only be tested in a longitudinal setting and by including more severe PD populations, with specific symptomatic subgroups.

Furthermore, the present systematic review has identified several knowledge gaps and important issues that need to be addressed by future neuroimaging research using task-based paradigms in PD patients. There is a need for future studies that should include patients at a later stage in the disease, as well as longitudinal investigations of brain activity in general, and cerebellar activity in particular. This will shed more light on how the pathology progresses and compensatory mechanisms unfold, as well as how these will impact the functional organization of the cerebellum in response to specific cognitive and motor demands. There is also a paucity of studies investigating the functional changes and reorganization of brain activity in PD in relation to pharmacological management of the disease over time. Finally, patient heterogeneity remains another important challenge that could be addressed by undertaking a better stratification of patients based on their disease pathology and symptomology, though understandably this may pose a challenge due to practical difficulties of including patients with a more advanced clinical state.

In conclusion, our study provides a review of task-based neuroimaging studies in PD with a focus on the functional specificity of the cerebellum. We show that the cerebellum in PD patients are hyperactive compared to healthy controls, irrespective of the task. We also show that cognitive functioning in PD is linked to the more recently developed cerebellar regions (i.e., Crus I and II, as well as lobule VI and VIII). In contrast, we also show that a lack of activity in motor related regions (lobule VI and vermal VII) is associated with motor symptoms severity. Together, these findings provide the first ever quantitative assessment of functional cerebellar involvement in PD patients, and importantly link various clinical aspects of the disease with specific sub-regions of the cerebellum.

## Data Availability Statement

All datasets generated for this study are included in the article/Supplementary Material.

## Author Contributions

LS contributed with the conception, design, analyses, interpretation, and write-up of the first draft of the manuscript. OL contributed with the scientific input, analyses, interpretation, and wrote sections of the manuscript. JD contributed with the interpretation and scientific input. All authors contributed actively in revisions of the manuscript, as well as approving the final, submitted version.

### Conflict of Interest

The authors declare that the research was conducted in the absence of any commercial or financial relationships that could be construed as a potential conflict of interest.
